# Luteinizing hormone supplementation in controlled ovarian stimulation: the Iran Delphi consensus

**DOI:** 10.3389/frph.2024.1397446

**Published:** 2024-05-09

**Authors:** Saghar Salehpour, Ashraf Aleyasin, Ashraf Moini, Nezhat Mousavifar, Nasresfahani Mohammadhossein, Sedighe Abdollahi Fard, Sanuiefarimani Marzie, Mahboubeh Mohammadzadeh, Robert Fischer

**Affiliations:** ^1^ Obstetrics and Gynecology Department, Shahid Beheshti University of Medical Science, Tehran, Iran; ^2^ Obstetrics and Gynecology Department, Tehran Medical Science University, Tehran, Iran; ^3^ Department of Gynecology and Obstetrics, Arash Women's Hospital, Tehran University of Medical Sciences, Tehran, Iran; ^4^ Department of Endocrinology and Female Infertility, Reproductive Biomedicine Research Center, Royan Institute for Reproductive Biomedicine, ACECR, Tehran, Iran; ^5^ Breast Disease Research Center (BDRC), Tehran University of Medical Sciences, Tehran, Iran; ^6^ Armaghan Infertility Center, Mashhad Medical Science University, Mashhad, Iran; ^7^ Animal Biotechnology Department, Reproductive Biomedicine Research Center, Royan Institute for Biotechnology, ACECR, Isfahan, Iran; ^8^ Obstetrics and Gynecology Department, Alzahra Hospital, Tabriz, Iran; ^9^ IVF Department, Hamedan Medical Science University, Hamedan, Iran; ^10^ Fertility Department, Merck Serono Middle East, Dubai, United Arab Emirates; ^11^ IVF Unit, Fertility Center Hamburg, Hamburg, Germany

**Keywords:** Delphi consensus, ovarian stimulation, ART, LH supplementation, Iran, expert

## Abstract

**Introduction:**

Numerous consensus documents worldwide address luteinizing hormone (LH) supplementation in controlled ovarian stimulation, yet to the best of our knowledge, only one consensus paper has been published in the Arab region. This study presents a Delphi consensus by seven Iranian infertility experts, offering real-world clinical perspectives. The aim was to develop evidence-based opinions on LH's role alongside FSH in various aspects of assisted reproductive technology (ART), including LH levels, monitoring, r-hLH use, and suggested activity.

**Methods:**

Employing the Delphi consensus approach, the Iran consensus unfolded in three steps. In Step 1, eight out of 10 statements gained approval, while two unclear statements were removed. In Step 2, the 20-member extended panel voted on the remaining eight statements.

**Results:**

Only one (statement 3) lacked consensus (55% agreement), prompting a modification. The revised statement (noted as statement 3′) obtained an 83% agreement.

**Discussion:**

The clinical perspectives included in this consensus complement clinical guidelines and policies that help further improve treatment outcomes, especially for patients with FSH and LH deficiencies.

## Introduction

Follicle-stimulating hormone (FSH) and luteinizing hormone (LH) are gonadotropins secreted by the pituitary gland under the pulsatile stimulus of gonadotropin-releasing hormone (GnRH) (1). Luteinizing hormone (LH) and follicle-stimulating hormone (FSH) play a complementary role in follicle development and ovulation. FSH initiates follicular growth, while LH acts at the follicle growth level, contributing to follicle maturation, fertilization and embryo quality (2). It affects the endometrium by promoting the decidualization of endometrial stromal cells and embryo implantation (3). Thus, a decrease or deficiency in the production or action of these gonadotrophins might compromise gametogenesis and gonadal steroid production, thereby reducing both female fertility and outcomes of medically assisted reproduction (MAR) (4, 5).

While the decrease in LH and FSH levels has been somehow extensively studied in the literature, their lack of action has been less documented (4, 6). LH and FSH deficiency may be congenital or acquired, functional/reversible, or permanent and may exhibit different degrees of severity. Several contributing factors have been identified and may explain the deficiency, including variability and impairment in gonadotropin-releasing hormone (GnRH) frequency, amplitude peaks and pulses, genetic variants in genes coding the gonadotropins and their receptors, and altered signaling pathways (7–9). Other identified demographic and clinical factors may also contribute to gonadotropin deficiency, such as advanced age, comorbidities (e.g., diabetes, thyroid disorders, eating disorders, excessive exercise, and tumors and related treatments), the use of contraceptive pills (10–15).

Several consensus documents have been developed around the globe regarding LH supplementation in controlled ovarian stimulation (16–18). Nevertheless, and to the best of the authors' knowledge, only one consensus paper has been published in the Arab region (19). In particular, a Delphi consensus provides a real-world clinical perspective from several experts, contributing to improved patient management and follow-up within a patient-tailored strategy (20, 21). In that perspective, seven Iranian experts in infertility management gathered to discuss and develop evidence-based opinions and statements regarding the LH role when co-administered with FSH in several aspects of assisted reproductive technology (ART).

## Consensus methodology and inclusion criteria for the panel of experts

**Figure 1:**
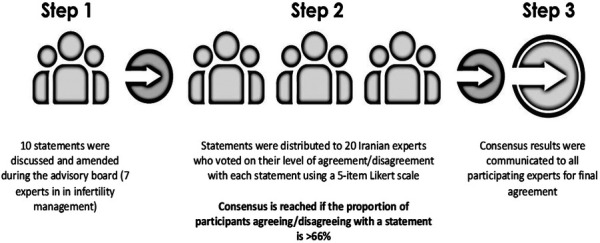
Consensus development methodology.

The Iran consensus was developed according to the Delphi consensus methodology and was achieved over three steps (Figure 1).

### Step 1

A panel of seven infertility experts, each affiliated with distinct medical universities based in Tehran, Hamadan, Isfahan, and East Azerbaijan province in Iran, were gathered with a scientific coordinator from Germany, an active member of the American Society of Reproductive Medicine, and a founding member of the European Society of Human Reproduction, for an interactive group discussion regarding ten statements proposed by the scientific coordinator and supported by updated references (Table 1). Statements were drafted, discussed, and amended by the experts' committee, when necessary, according to the available scientific evidence and current clinical practice.

**Table 1:** Statements approved by the scientific board.

Statement	Level of agreement	Details of the statement	Reference
Statement 1	90%	Following administration of gonadotropin-releasing hormone (GnRH) agonists/antagonists, a transient severe LH deficiency can occur in some patients.	(4, 22–26)
Statement 2	85%	FSH and LH deficiency in patients with associated risk factors (e.g., advanced maternal age, metabolic disorders and eating disorders) can be exacerbated by the use of GnRH analogs.	(4, 22–26)
Statement 3	55%	In antagonists’ protocol, LH levels should be monitored during the stimulation cycle after starting the antagonist to identify severe LH deficiency. In GnRH agonist protocols, LH levels should be monitored after completing downregulation before the start of ovarian stimulation.	(23, 27)
Statement 3′	83%	In antagonist protocols, LH level could be monitored during the stimulation cycle after starting the antagonist to identify sever LH deficiency. In GnRH agonist protocols, LH level could be monitored after downregulation before the start of ovarian stimulation.	(23, 27)
Statement 4	100%	Low levels of E2 in relation to the follicular response may indicate low levels of LH activity during stimulation with recombinant human FSH (r-hFSH) monotherapy	(4)
Statement 5	100%	Some polymorphisms of FSH, LH, and their receptors will affect gonadotropin bioactivity and their response during ovarian stimulation, resulting in a lower-than-expected oocyte yield.	(9, 27)
Statement 6	100%	The use of r-hLH with r-hFSH compared to r-hFSH monotherapy will improve the ongoing pregnancy rate in some groups of low prognosis patients (Poseidon GI, GII and GIV).	(4, 28–31)
Statement 7	100%	LH and Human chorionic gonadotropin (hCG) are characterized by specific molecular and biochemical features; they interact with distinct binding sites on the same receptor, and the dissociation rates from these sites are lower for hCG compared with LH. r-hLH has a shorter terminal half-life. Downstream effects of gonadotropins’ signaling consist of LH-related proliferative and anti-apoptotic signals, vs. high steroidogenic potential and pro-apoptotic activity of hCG *in vitro*.	(18, 32–34)
Statement 8	95%	LH modulates various signaling molecules involved in implantation namely, leukemia inhibiting factor, colony-stimulating factor-1, interleukin-1, integrins, glycodelin and mucin 1, and may improve endometrial receptivity and implantation.	(4)

### Step 2

The statements were then distributed to 20 infertility experts before the voting session, who voted on their level of agreement or disagreement with each statement using a 5-point Likert scale: 1 (absolutely disagree), 2 (disagree), 3 (agree), 4 (more than agree), and 5 (absolutely agree) (20, 35). Consensus was reached if the proportion of participants agreeing or disagreeing with a statement was >66%. Statements that did not reach consensus were updated and sent again for voting.

### Step 3

Based on the outcomes of Step 2, the revised statements were communicated to all participating experts for final agreement. The present manuscript was written based on the group discussion; it was reviewed by all experts, who incorporated their experience regarding the role of LH in ART.

## Results of the consensus and recommendations

**Figure 2:**
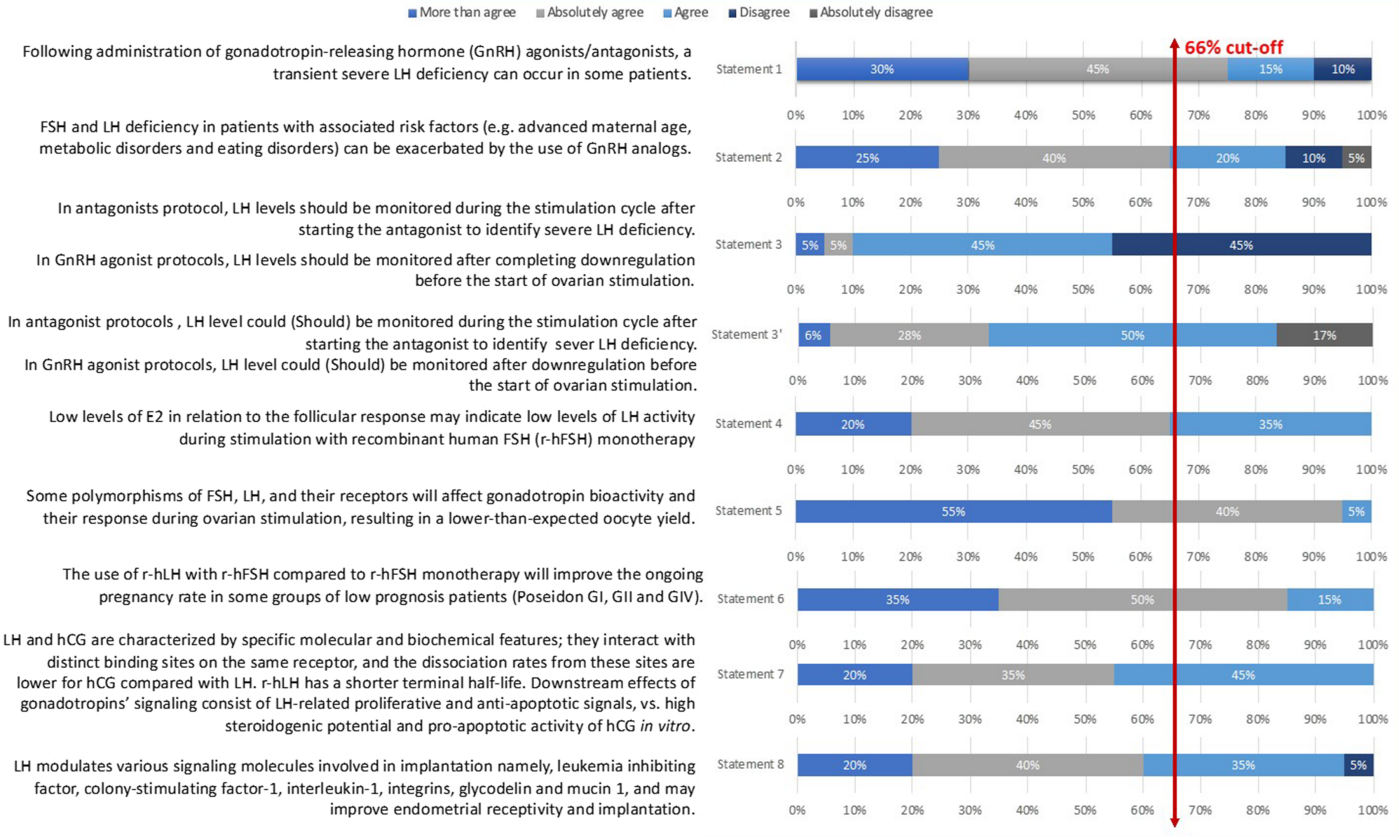
Level of agreement/disagreement on each statement.

In Step 1, eight out of ten statements were approved after discussion and modification. Two redundant or deemed unclear statements were removed. The remaining eight statements were then voted in Step 2 by the 20-member extended panel. Only statement 3 did not reach a consensus (55% agreement); thus, the committee suggested a modification. This new statement (noted statement 3′) obtained an 83% agreement afterward. Details are presented in Table 1 and Figure 2.

Statement 1: Following the administration of gonadotropin-releasing hormone (GnRH) agonists/antagonists, a transient severe LH deficiency can occur in some patients. This statement reached a 90% level of total agreement among the extended expert panel (Figure 2).

## Discussion statement 1

GnRH agonists and antagonists are used during ovarian stimulation (OS) to enable the clinical retrieval of the maximum number of oocytes. They can induce a transient deficiency in LH and FSH, effectively preventing premature ovulation. The analogs exert different mechanisms of action on endogenous gonadotropins, causing either a gradual (GnRH agonists) or an abrupt (GnRH antagonist) suppression (4, 36, 37). Whether with agonists or antagonists, residual LH levels are usually enough to support steroidogenesis and allow OS following the administration of recombinant FSH (r-FSH); however, a severe deficiency can occur in some patients (4, 22). In GnRH agonist cycles, due to the reduced gonadotropin production, a severe deficiency can be observed when the LH levels drop below a threshold value (ranging from < 1.5–0.5 IU/L LH, according to the literature) (28, 38, 39). Several studies demonstrated that standard long GnRH agonist protocols followed by an OS with r-FSH led to significant severe LH deficiency, seen in up to almost 50% of normogonadotropic women (39, 40). Such observations were associated with higher early pregnancy loss (39) and lower live birth rates (40). One hypothesis that can be put forward to explain such results is that the abrupt drop in LH levels during OS might be related to lower E2 production by the follicles and, consequently, lower circulating E2 levels (4, 22).

Although less frequently observed, LH suppression can also be documented in GnRH antagonist protocols, with detrimental effects on the quality and quantity of eggs (41, 42), thus altering treatment outcomes. In 2014, Kol reported that LH was over-suppressed in 26% of women, and these patients had a significantly lower increase in E2 during the first 24 h after antagonist administration compared to women who were not over-suppressed. Nevertheless, some authors suggested that severe suppression is not observed in all patients but only in some subgroups, such as, but not limited to, patients with advanced maternal age. Moreover, negative reproductive outcomes were postulated to result from the magnitude of suppression over time vs. the baseline rather than a drop in the absolute LH levels, as previously mentioned (43). The addition of r-LH seems to reverse the effect of the reported deficiency (4, 44).

Statement 2: FSH and LH deficiency in patients with associated risk factors (e.g., advanced maternal age, metabolic disorders, and eating disorders) can be exacerbated by the use of GnRH analogs.

This statement had an 85% level of total agreement among the extended expert panel (Figure 2).

## Discussion statement 2

Aging, metabolic diseases (including diabetes or thyroid disorders), and eating disorders (including obesity and anorexia) are widely recognized to significantly influence gonadotropin secretion and action, mainly by affecting the hypothalamic-pituitary axis (4, 45). Hence, FSH and LH deficiencies already observed in these patients, especially women with advanced maternal age, seem to be exacerbated by the administration of GnRH analogs, probably due to the transient gonadotropin deficiency (Bosch et al. 2021). In that context, identifying and treating the underlying disorders is paramount to restoring reproductive function and improving stimulation outcomes (4, 11, 46). Thus, several reports, including women aged 35 to 40 years old, demonstrated that the r-hFSH:r-hLH co-supplementation led to higher implantation (and oocytes maturation) and birth rates as compared to r-hFSH monotherapy (4, 47–49)

Statement 3: In antagonist protocols, LH levels could be monitored during the stimulation cycle after starting the antagonist to identify severe LH deficiency. In GnRH agonist protocols, LH levels could be monitored after downregulation before the start of ovarian stimulation.

The statement that was voted during the Step 1 expert meeting consisted of the following: “In antagonists’ protocol, LH levels should be monitored during the stimulation cycle after starting the antagonist to identify severe LH deficiency; In GnRH agonist protocols, LH levels should be monitored after completing downregulation before the start of ovarian stimulation” (Statement 3 in Table 1 and Figure 2). Only 1 of 7 (14%) experts disagreed on it during the voting session. Nevertheless, only 55% of the Step 2 expert board agreed with the statement as stated earlier; therefore, it did not reach a consensus. The committee of seven Iranian experts then suggested the following changes: “In antagonist protocols, LH levels could be monitored during the stimulation cycle after starting the antagonist to identify severe LH deficiency; In GnRH agonist protocols, LH levels could be monitored after downregulation before the start of ovarian stimulation” arguing that LH monitoring is not mandatory but could be used to improve the quality of the cycle and clinical outcomes (Statement 3′ in Table 1 and Figure 2). The final statement was then resent to the 20 experts for voting and reached an 83% level of total agreement.

## Discussion statement 3

FSH and LH are both essential components for folliculogenesis; this is the concept of “two cell–two gonadotropin” described in the literature (50). By stimulating the theca cells in the ovary, LH plays a critical role in androgen production, thus facilitating estradiol production and FSH activity (23).

Therefore, determining LH levels is recommended if a severe LH deficiency is anticipated. Hence, in antagonist protocols, some subgroups of patients with LH drop (as described in Statement 1) would require r-hLH supplementation from the beginning of the cycle; these include women at an advanced reproductive age (36–39 years old) and women with adequate pre-stimulation ovarian reserve parameters and an unexpected hypo-responders to r-hFSH monotherapy (27). Moreover, if LH levels remain low, they reflect a state of severe deficiency, and patients might not have an adequate response to GnRH agonists.

In GnRH agonist protocols, LH level measurements could rather be performed before ovarian stimulation. A systematic review (27) noted a severe mid-follicular LH deficiency in 7%–48% of normogonadotropic women undergoing OS, which might affect the ovarian response to r-hFSH monotherapy. It also highlighted controversial results related to pregnancy outcomes, with some studies reporting early pregnancy loss and reduced fertilization rates when LH levels were 0.5–0.7 IU/L (39, 51), while others did not observe any difference (27, 38, 52). Further research is necessary to clarify these findings. In Iran, LH levels are not measured routinely; however, in the case of FSH and LH deficiencies due to GnRH analogs, the 5 IU/L is considered a deficiency.

Of note, evidence suggested that the absolute LH serum level might not correctly reflect LH deficiency but rather the magnitude of suppression over time compared to the baseline (43). Therefore, when investigating LH deficiency, clinicians should focus on exploring the difference in LH levels before and after the administration of GnRH analogs (delta) rather than relying solely on a simple cut-off value.

Statement 4: Low levels of E_2_ in relation to the follicular response may indicate low levels of LH activity during stimulation with recombinant human FSH (r-hFSH) monotherapy. This statement had a total agreement from the extended panel (100%; Figure 2).

## Discussion statement 4

During the first meeting, experts stated that, in the Iranian practice, the measure of E_2_ levels might not be feasible due to cost constraints. Nevertheless, regardless of this economic issue, estradiol levels should be monitored 2–3 times during the cycle in ovarian stimulation protocols, and for poor responders, testing more than three times may be necessary. Evidence from the literature suggested that low E_2_ levels might be considered relevant endocrine endpoints for LH and FSH deficiencies (4). E_2_ could reflect the effect of LH on steroidogenesis in both theca and granulosa cells; thus, low levels of E_2_ that do not match the size and number of follicles (E_2_/oocyte ratio) might suggest low levels of LH activity during OS.

Statement 5: Some polymorphisms of FSH, LH, and their receptors will affect gonadotropin bioactivity and their response during ovarian stimulation, resulting in a lower-than-expected oocyte yield. This statement had a 100% level of agreement among the extended expert panel (Figure 2).

## Discussion statement 5

Cumulative evidence highlighted interindividual differences in ovarian response to gonadotropin stimulation related to polymorphisms in genes encoding for the gonadotropins or their receptors (4, 7, 53). A recent systemic review with meta-analysis (53) highlighted that several single nucleotide polymorphisms (SNP), especially in the gene encoding the FSH receptor, *FSHR*, have been shown to modulate ovarian response and are among the best candidates to be selected as markers to predict individual response to OS.

The SNP *FSHR* rs6166 (c.2039G > A; p.Asn680Ser) was extensively studied in the literature. Studies have shown that this polymorphism could also affect OS. Indeed, a recent Delphi consensus related to this polymorphism reported that the Ser/Ser genotype was associated with a reduced sensitivity of the FSHR to exogenous FSH (7). Moreover, patients carrying two copies of the variant Ser allele required higher amounts of gonadotropin during OS, had higher basal levels of FSH, and produced fewer oocytes and fewer metaphase II oocytes in response to OS than Asn/Asn or Asn/Ser patients (7). Interestingly, a randomized controlled trial showed that increasing the FSH dose might revert this reduced sensitivity (54).

The rs6165 (c.919G > A; p.Thr307Ala) polymorphism is another SNP in the *FSHR* in strong linkage disequilibrium with rs6166: patients with the AA genotype had a significantly higher number of retrieved oocytes, a higher number of metaphase II oocytes, and necessitated a shorter duration of gonadotropin stimulation as compared to the other groups of patients (53).

The *FSHR* rs1394205 (c.-29G > A) polymorphism, located in the promoter region, has also been extensively studied and suggested as a critical marker to predict ovarian response in assisted reproductive technology (9). Studies in specific ethnic populations have demonstrated that homozygote AA patients had lower ovarian sensitivity and produced significantly fewer oocytes, thus necessitating significantly higher FSH consumption to achieve an adequate OS as compared with GA and GG patients (53).

A recent multicenter, multinational prospective study (2016–2019), which enrolled 366 predicted normal responders from Vietnam, Belgium, and Spain, yielded controversial results regarding genetic susceptibility in response to OS. Thus, the authors failed to reproduce the previously published genetic correlations since none of the studied SNPs (rs6165, rs6166, and rs1394205) was significantly associated with the late follicular phase serum progesterone or estradiol levels (55).

Other genetic variants in the gene encoding the FSH beta subunit (*FSHB* rs10835638; c.-211G > T), the luteinizing hormone b-chain (LHB), and the LH/choriogonadotropin receptor (LHCGR) might also affect ovarian stimulation, but more evidence is required to confirm their implication (4, 7, 53).

Statement 6: The use of r-hLH with r-hFSH compared to r-hFSH monotherapy will improve the ongoing pregnancy rate in some groups of low prognosis patients (Poseidon GI, GII and GIV). This statement had a total agreement (100%) from the extended panel (Figure 2).

## Discussion statement 6

Several studies have explored the role of r-hLH in ovarian stimulation for ART. A systematic review from 2018 concluded that rhLH supplementation might be beneficial, particularly in two groups of patients, i.e., (1) women with adequate prestimulation ovarian reserve parameters (Antral follicle count- AFC ≥5, Anti-Mullerian Hormone- AMH ≥ 1.2 ng/mL) and an unexpected hyporesponse to r-hFSH monotherapy (unexpected poor or suboptimal ovarian response; Poseidon Groups 1 (age < 35 years) and 2 (age ≥ 35 years) and (2) women with advanced maternal age (35–39 years old), including those from the Poseidon Group 4 (Age ≥ 35 years, with poor ovarian reserve prestimulation parameters: AFC <5 & AMH < 1.2 ng/mL) (27, 56). In all other cases, the results remain controversial and require further research to confirm the need for rhFSH supplementation (27). This supplementation with rhLH seems to have an added value for pregnancy outcomes. Indeed, a literature review with metanalysis, including 12 randomized control trials, showed that using r-hLH with r-hFSH as compared to the hFSH alone yielded higher pregnancy and implantation rates, especially in GnRH agonist protocols, while evidence is still debatable with GnRH antagonist protocols (6, 44). A recent *in vitro* study tested whether the addition of LH to FSH affects the response of granulosa lutein cells collected from poor-, sub-, and normoresponder women undergoing MAR. These cell lines are an excellent model to evaluate the co-administration of both LH and FSH since they express receptors for the two gonadotropins. Primary endpoints consisted of cAMP and progesterone production. The results showed that LH addition in the poor-responder and sub-responder groups enabled some recovery of the FSH-induced cAMP and progesterone production since these were similar to those observed in normoresponder women (57).

Statement 7: LH and hCG are characterized by specific molecular and biochemical features; they interact with distinct binding sites on the same receptor, and the dissociation rates from these sites are lower for hCG compared with LH. r-hLH has a shorter terminal half-life. Downstream effects of gonadotropins' signaling consist of LH-related proliferative and anti-apoptotic signals, vs. high steroidogenic potential and pro-apoptotic activity of hCG *in vitro*.

Statement 7 had a 100% level of agreement among the extended expert panel (Figure 2).

## Discussion statement 7

Several studies, systemic reviews, and meta-analyses discussed comparatively the molecular and biochemical features of LH and hCG. LH and hCG consist of heterodimeric glycoproteins that share a common alpha-subunit but a distinct beta-subunit. Due to these similarities, both hormones can bind to the same receptor, i.e., the LH chorionic gonadotropin receptor (LHCGR), but their pharmacokinetic characteristics and molecular responses are somewhat different (5, 18, 32). Hence, r-hLH has a shorter terminal elimination half-life, estimated to be around 10 h, as compared to the 28–31 h for the hCG after intravenous administration (18, 58, 59). Moreover, signaling pathways following the receptor stimulation are distinct as well. LH works as a partial agonist for progesterone, with a proliferative and antiapoptotic action. It exerts its action mainly through the activation of kinases (extracellular signal-regulated kinase ½ [pERK1/2] and protein kinase B (AKT)-dependent pathways). hCG, oppositely, displays a notable steroidogenic and proapoptotic potential, along with a decreased proliferation of granulosa cells, mainly via the upregulation of the cAMP/protein kinase A (PKA) and caspase-3 pathways. However, this apoptotic effect of hCG seems to be masked by the action of estrogen *in vivo* (5, 18).

Statement 8: LH modulates various signaling molecules involved in implantation, namely leukemia inhibiting factor, colony-stimulating factor-1, interleukin-1, integrins, glycodelin, and mucin 1, and may improve endometrial receptivity and implantation. This last statement had a 95% total level agreement by the extended expert panel (Figure 2).

## Discussion statement 8

Evidence highlighted that LH receptors are expressed in the human endometrium (epithelial and stromal cells) and that LH can affect uterine receptivity independently of ovarian function (4, 60). Moreover, a study has shown that patients with low endogenous LH levels would have a disturbed endometrial maturation that can be rescued by a mid-cycle administration of exogenous hCG or LH, which would stimulate LH receptors (60). Several factors seem to be involved in the endometrial function that enables the implantation process, including leukemia inhibiting factor, colony-stimulating factor-1, interleukin-1, integrins, glycodelin, and mucin 1 (MUC1) (4, 60, 61)

## Limitations and strength

The statements only represent the collective opinion of the experts included. Moreover, the consensus was reached based on references selected by the scientific adviser who might have omitted relevant information. Furthermore, not all statements reached 100% agreement, with some statements reaching consensus even though some participants disagreed with them. Some of these statements might also evolve with new evidence emerging from randomized controlled studies. Nevertheless, and despite these limitations, this Delphi consensus provides a real-world clinical perspective on the LH supplement in COS from group of Iranian Expert.

## Conclusion

The clinical perspectives included in this consensus supplement clinical guidelines and policies that help to further improve treatment outcomes especially patients with FSH & LH deficiency.

## Data Availability

The original contributions presented in the study are included in the article/Supplementary Material, further inquiries can be directed to the corresponding author.
